# Improvements in Performance Analysis of Photovoltaic Systems: Array Power Monitoring in Pulse Width Modulation Charge Controllers

**DOI:** 10.3390/s19092150

**Published:** 2019-05-09

**Authors:** Gabino Jiménez-Castillo, Francisco José Muñoz-Rodríguez, Catalina Rus-Casas, Pedro Gómez-Vidal

**Affiliations:** 1 Department of Electronic and Automatic Engineering, Universidad de Jaén, Campus Lagunillas, 23071 Jaén, Spain; gjimenez@ujaen.es (G.J.-C.); crus@ujaen.es (C.R.-C.); 2 Centre for Advanced Studies in Energy and Environment CEAEMA, Universidad de Jaén, 23071 Jaén, Spain; pvidal@ujaen.es; 3 Department of Electricity Engineering, Universidad de Jaén, Campus Lagunillas, 23071 Jaén, Spain

**Keywords:** photovoltaic systems, pulse width modulation, monitoring, array power

## Abstract

Various challenges should be considered when measuring photovoltaic array power and energy in pulse width modulation (PWM) charge controllers. These controllers are frequently used not only in stand-alone photovoltaic (SAPV) systems, but may also be found in photovoltaic (PV) self-consumption systems with battery storage connected to the electricity grid. An acceptable solution may be reached using expensive data acquisition systems (DASs), although this could be generally disproportionate to the relatively low cost of SAPV systems. Therefore, the aim of this paper is to develop new and effective monitoring techniques which will provide the PV array direct current (DC), output power (P_A,dc_), and PV array DC output energy (E_A_), thus avoiding the use of sophisticated DASs and providing high accuracy for the calculated parameters. Only transducers and electronic circuits that provide the average and true rms values of the PWM signals are needed. The estimation of these parameters through the aforementioned techniques showed high accuracy for both series and shunt PWM battery charge controllers. Normalized root mean square error (NRMSE) was lower than 2.4%, normalized mean bias error (NMBE) was between −1.5% and 1.1%, and mean absolute percentage error (MAPE) was within 1.6%.

## 1. Introduction

Off-grid and mini grid systems with renewable energy have gained a competitive advantage over the last five years, and this change is expected to accelerate in the future [1]. More than half of the populations of developing countries live in rural regions [2], where photovoltaic solar energy can play a key role in rural electrification. Therefore, a stand-alone photovoltaic (SAPV) system could be a good solution for remote places [3,4,5]. It is estimated that around 133 million people accessed lighting and other electricity services using off-grid renewable energy solutions in 2016; 24 million of them were using a solar home system (SHS) [6]. In 2013, it was estimated that there were more than six million SHS systems [7]. SHS systems are low cost SAPV systems with very low array power. In addition, in recent years, the reduction in price of PV modules (by 81%) and batteries since the end of 2009 [8] has led to a growing interest in “living off-grid” or “leaving the grid” [9].

Photovoltaic (PV) systems that are not connected to the public grid can be defined as SAPV or off-grid PV systems, and they are designed to cover electricity needs. Overall, the basic elements of SAPV systems are a PV generator, a battery charge controller (BCC), an energy storage system, loads or appliances, and an inverter is also used when there are alternating current (AC) loads. Battery charge controllers regulate the PV array, the load, and the battery current in order to prevent battery over-discharging and overcharging. BCCs are often categorized by the strategies used to regulate the charge from the PV generator to the energy storage system [10,11,12,13]. Pulse width modulation (PWM) and maximum power point tracking (MPPT) are the most widely used controllers in the SAPV industry [14].

A SAPV system is solely responsible for supplying the electric power to meet the load needs, and it operates independently of the electric utility grid. For these reasons, adequate performance must be ensured. The real operation of PV systems depends on local uncertain weather conditions, such as relative dust, ambient temperature, relative humidity, global solar radiation, etc. [15]. An inspection of functioning systems under real climate conditions showed the technical issues, and also a way to improve the systems [16]. Photovoltaic energy harvesting is also influenced by the degradation and maintenance of the system. After the installation of PV systems, a maintenance service is needed in order to supervise their operation and to detect possible malfunctions. Maintenance operations play an important role in analyzing life expectancy and in evaluating the viability of PV systems [17], while preventive maintenance can be improved by monitoring systems [18]. It has been observed that in developing countries there is a lack of maintenance services, which necessitates a proactive program of monitoring and assistance [19]. The purposes of monitoring should be to provide useful performance information through the proper measurement of determined parameters.

Data acquisition systems are widely used for monitoring PV systems, where performance of the system has been evaluated under real conditions [20,21,22,23]. Operational monitoring of PV systems and analysis of the collected data have made continuous progress in recent years [24,25,26]. Moreover, user-friendly graphical interfaces (GUI) have improved the monitoring systems [27]. In this sense, data-acquisition systems can be managed and controlled by the GUI, which can be developed by software tools such as LabVIEW^®^, which allows real-time interface [28] and online real-time interface [29], or SCADA systems [30]. In addition, web-based applications are used to control and monitor photovoltaic systems, which allow the distribution of data to remote users [31]. Open hardware–software platforms are also used extensively as parts of photovoltaic monitoring systems [32], and they have also promoted the expansion of the monitoring systems [33]. A procedure for data acquisition systems has been designed with a microcontroller where Excel has been integrated by using Visual Basic program in order to plot and analyze the data in real time for Stand-Alone Systems [34]. Wireless photovoltaic monitoring systems have been designed and tested [35], where the monitoring system can be managed by a central computer which is connected to sensors by RF modules [36].

However, due to the cost of monitoring systems, these improvements can rarely be applied to small and medium SAPV systems. As can be found in practice, small PV systems are not regularly checked [37], such as professional SAPV systems or small grid-connected PV systems. Unfortunately, problems associated with technical failures in small and medium-sized PV systems are widely documented [38]. In West Africa, many projects have been implemented, ranging from solar powered borehole pumps to energy supply systems in hospital emergency departments. However, most of them have been left to deteriorate without maintenance, cleaning, and repairs [39]. A study of approximately 135 remote households in rural Thailand has found that 50% of the main components of their photovoltaic systems were damaged [40]. In Fiji, most of the 250 solar home systems (about 80%) that were installed under the Vunivaou solar home system project were found not to be functioning [41].

SAPV system monitoring can be expensive, depending on the parameters to be monitored, and can subsequently be tedious due to a lack of infrastructure, but helps to improve preventative maintenance, which, in turn, reduces the response time in the case of a system failure [18]. Moreover, proper monitoring can find potential faults and evaluate whether the PV system is still operating as expected [42]. For these reasons, the International Standard IEC Standard 61724-1 was developed by the International Electrotechnical Commission (IEC) technical committee [42]; it is a new edition of IEC 61724 that was published in 1998. However, the updated Standard 61724-1 and the previous edition are specially intended for grid-connected systems, and may not provide affordable solutions that take into account the particularities of SAPV systems [11,12].

## 2. Background and Objectives

As mentioned above, there are two main types of battery charge controller, the PWM and MPPT. The latter is usually an electronic DC/DC converter that operates the PV array at its maximum power point along the P–V curve. As the output power of MPPT BCC is given by DC currents and voltages, there may not be any difficulties when measuring them.

On the other hand, PWM battery charge controllers provide asymmetric waveforms. A PWM battery charge controller adjusts the duty factor (d_f_), defined as the ratio of the pulse duration to waveform period [43], of the current signal that comes from the photovoltaic system and goes to the battery. The duty factor depends on the battery state-of-charge (SOC). These types of techniques, which are applied in lead–acid batteries, can accelerate the battery recharge and increase the life cycle performance of low-maintenance batteries [44,45]. However, between the monitoring system cost and the accuracy of the monitored data, proper monitoring of PV systems that use PWM techniques is a challenge.

Depending on the PV system size and user objectives, the required accuracy of the monitoring system is selected [42], where medium or basic accuracy may be the best option for SAPV systems. Monitoring systems with medium and basic accuracy should take one minute between acquired samples; thus, the sampling interval should be one minute maximum. However, PV systems that use PWM battery charge controllers may require a special measurement or monitoring equipment to accurately measure the provided signals [46]. Moreover, the sampling frequency of the signals should conform to the Annex A of the IEC 61724-1, which recommends that a sampling interval should be lower than the waveform period divided by two hundred in order to reconstruct the signal with an accuracy of 1% [42]. It should be highlighted that the main frequency in series and shunt charge controllers has a value from ten to hundreds of Hertz [11].

A further issue to consider in SAPV systems that use PWM battery charge controllers is the error that can be created when sampled data are used to calculate other parameters, such as the current, voltage [11], power, or energy [12]. Nevertheless, this error could be avoided if the data acquisition system (DAS) obtained synchronized and simultaneous current and voltage samples at the required sampling frequency. A DAS consists of all the components required for the monitoring process: sensors, transducers, electronic circuits, data acquisition devices or data acquisition boards, computer, etc. Therefore, synchronized and simultaneous samples are possible to obtain with advanced equipment, although this technique is rather costly, taking into account that SAPV systems, in most cases, are relatively low cost [47]. Therefore, a change in approach is needed to address the aforementioned challenges without using sophisticated and expensive monitoring systems. In this way, an alternative method, which addresses the performance analysis of SAPV systems with PWM charge controllers and batteries, was developed [12]. This method is focused on the monitoring of currents instead of power, which are used to calculate charge parameters. Charge parameters are considered as an adaptation of the energy parameters provided by the IEC Standard 61724. This method estimates the charge parameters in SAPV systems with a PWM battery charge controller each minute, without using complex and simultaneous sampling. Moreover, the method recommends using DC Hall effect transducers to measure battery, loads, and array currents, instead of shunt resistors [11].

Nevertheless, this method does not provide a solution which meets all the requirements of IEC Standard 61724-1, as it does not allow estimation of the power parameters as required, making it difficult to compare the performances of SAPV systems with different types of battery charge controller, the PWM and MPPT. In the latter, the performance analysis was carried out using energy parameters. Moreover, this method cannot obtain pulse parameters, such as low and high states or the duty factor of PWM waveforms. The duty factor can be used to estimate battery losses caused by a high SOC. Battery losses can be considered as structural losses of the SAPV system, which may be confounded by other energy losses. Moreover, once these types of losses are quantified, it will be easier to estimate other energy losses. Therefore, the aim of this paper, and herein lies its novelty, was to develop new, simple, and affordable techniques to monitor the array power in SAPV systems with PWM charge controllers, which will overcome the aforementioned drawbacks related to the different monitoring methods found in the literature. The new monitoring techniques will provide PV array DC output power (P_A,dc_), the duty factor (d_f_), and PV array DC output energy (E_A_) without using sophisticated DASs, where data acquisition devices (DAQ) with synchronization and high sampling frequency are not needed. Furthermore, a great deal of collected data are generated, and more sophisticated DAQ devices are needed when a high sampling frequency of the measurement is required. In this sense, the proposed technique allows a sampling interval and a recording interval of one minute; therefore, there may be extra storage in the DAQ device memory, as the data generated may be extremely low compared with the first solution.

The proposed monitoring techniques, along with an unsophisticated DAQ device, require the use of sensors, transducers, or simple electronic circuits that provide the average and true rms values of the PWM signals. Moreover, from these values, different parameters may be estimated such as the d_f_, low and high states of the PWM signals, that will not only allow estimation of P_A,dc_ and E_A_, but will also permit the reconstruction of the PWM signals.

The paper is focused on developing different equations that allow estimation of the aforementioned parameters, P_A,dc_ and E_A_, from only two of these three parameters: average and true rms values, and the duty factor of the PWM array waveforms (i.e., array current and voltage waveforms). The equations, which is developed in the next section, have been obtained considering “clean” PWM waveforms. However, as shown later, the “real” waveforms provided by charge controllers may have overshoot, undershoot, ringing, and positive and negative transition durations, so it is necessary to check the validity of the proposed techniques in real operation, considering different values of the duty factor. This is the main objective of this paper: to develop new and simple monitoring techniques for SAPV systems with PWM charge regulators, and to validate them considering real data obtained from two SAPV systems with different types of PWM charge controllers (shunt and series). Once the aforementioned techniques are validated, the techniques shown here can be implemented in different ways using less sophisticated and complex DAQ devices, together with average and true rms transducers. Moreover, DAQ devices can be substituted by hardware–software platforms, e.g., a Mini USB Arduino Nano board and micro SD cards as a storage elements [48], an Arduino Mega board and two SD cards [49], or an Arduino UNO board which is connected to the computer [50]. The transducers can be substituted by electronic circuits designed ad hoc to obtain two of these three parameters: average, true rms, or duty factor values. The duty factor can also be obtained using a specific microcontroller with a software routine and peripherals. In the literature, different strategies of software routines for microcontrollers to measure the waveform d_f_ can be found [51,52]. Any of these different choices can provide P_A,dc_ and E_A_. However, the different ways to implement the method shown here are beyond the scope of this paper.

P_A,dc_ and E_A_ constitute very useful information with which to provide proper array performance, and therefore, to give adequate system performance. As is shown in IEC-61724-1, once E_A_ is estimated from P_A,dc_ , different yields can be obtained which indicate actual array operation relative to its rated capacity: PV array energy yield (Y_A_) and yield losses such as array capture losses (L_C_) and balance of systems losses (L_BOS_). In this sense, the estimation of many parameters, yields, and efficiencies depends on E_A_. Therefore, a proper estimation of this parameter will provide a correct estimation of the aforementioned parameters, and, therefore, proper system performance will be obtained. Moreover, it must be highlighted that only two parts are considered in a PV system: the array and the BOS (balance of system, i.e., the rest of the elements of the system apart from the array: charge controller, inverter, battery, loads, wires, protections). Therefore, the PV array constitutes a fundamental part of the SAPV system. If a proper system performance is to be achieved, it is necessary to develop proper array performance through P_A,dc_ and E_A_.

The manuscript proceeds with the mathematical background. Next, the new monitoring techniques to be used in SAPV are described. It is shown how to obtain P_A,dc_ and E_A_ parameters using a shunt and series PWM charge controllers. In Section 4, the experimental setup used to validate the aforementioned techniques is shown. Section 5 analyses the error obtained when estimating: the d_f_, high state of PV array current (i_A,H_), low state of PV array current (i_A,L_), high state of PV array voltage (v_A,H_), low state of PV array voltage (v_A,L_), and P_A,dc_. Furthermore, in this section, the statistical metric indicators related to E_A_ were calculated considering a daily reporting period. In order to quantify the error properly, the calculation of E_A_ was only applied when there is a PWM. Finally, conclusions are summarized in Section 6.

## 3. Methods

Battery charge controllers use different methods to regulate the current between batteries and PV array systems. The most common are the MPPT and PWM techniques. PWM techniques may be divided in turn into two categories: shunt and series.

**Figure 1:**
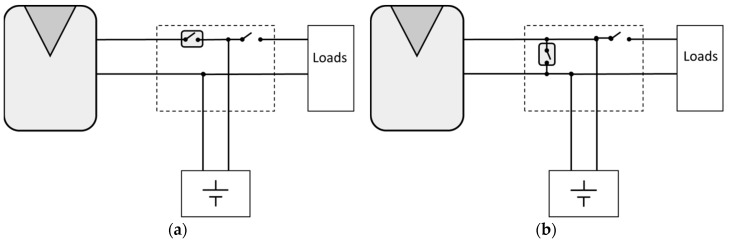
Simplified stand-alone photovoltaic (SAPV) schemes: (**a**) SAPV with a series pulse width modification (PWM) battery charge controller (**b**) SAPV with a shunt PWM battery charge controller.

Series PWM battery charge controllers (Figure 1a) regulate the flow of electricity switching the series element at a fixed frequency. They open the path between the battery and the PV array, which results in the current flow being interrupted. Shunt PWM battery charge controllers (Figure 1b) can be very similar to series charge controllers, but instead of opening the circuit, they short-circuit the PV array modules output through the shunt element.

**Figure 2:**
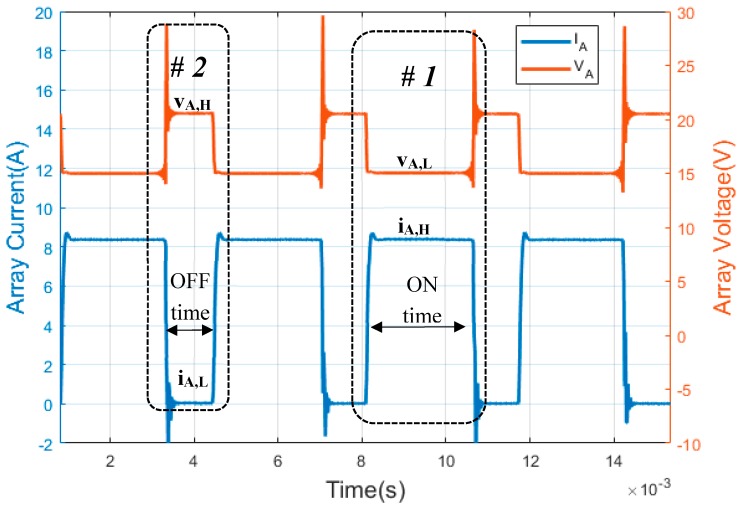
Waveforms of a series PWM battery charge controller. (Y-axis left) photovoltaic (PV) array current. (Y-axis right) PV array voltage. The PWM waveforms were obtained through the experiments in this paper.

**Figure 3:**
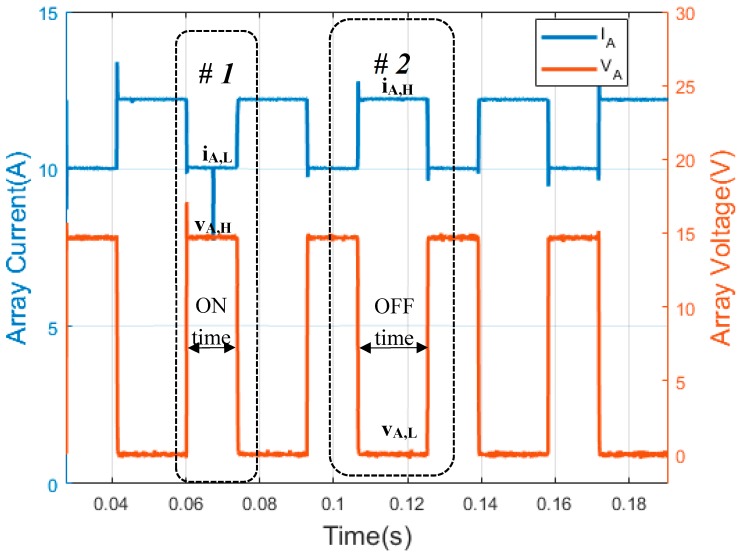
Waveforms of shunt PWM battery charge controller. (Y-axis left) PV array current. (Y-axis right) PV array voltage. The PWM waveforms have been obtained through the experiments in this paper.

As can be observed in Figure 2 and Figure 3, the two state levels that characterize each PWM signal must be defined (Table 1). For series PWM battery charge controllers (Figure 2) state level #1 (ON time) is defined when PV array current has a high state (i_A,H_). However, PV array voltage has a low state (v_A,L_); this state corresponds with the PV array and the battery being connected. On the other hand, the state level #2 (OFF time) is when there is no current in the PV array and the PV array has open circuit voltage. Therefore, a low state of PV array current (i_A,L_) and high state of PV array voltage (v_A,H_) are reached.

In these waveforms (Figure 2) the duty factor of the PV array current (d_f,i_) waveforms and the duty factor of the PV array voltage (d_f,v_) waveforms of SAPV with a series PWM battery controller can be expressed as follows:

(1) $$d_{f,v}=1-d_{f,i}$$

Figure 3 shows PV array waveforms of SAPV with a shunt PWM battery controller; Figure 3 (Y-axis left) shows the PWM array current waveform and Figure 3 (Y-axis right) illustrates the PWM array voltage waveform. As can be seen in these figures, the low state of PV array current (i_A,L_) is used to define the state level #1, where the PV array voltage has a high state (v_A,H_). On the other hand, OFF time is considered when the PV array is short-circuited. Thus, there is a short-circuit current in the PV array, and the voltage across the PV array is zero, i.e., the high state of PV array current (i_A,H_) and the low state of PV array voltage (v_A,L_), respectively. As with series PWM battery charge controllers, the duty factor of the PV array voltage waveforms (d_f,v_) and the duty factor of the PV array current waveform (d_f,i_) can be expressed as follows:

(2) $$d_{f,i}=1-d_{f,v}\;$$

The frequency of the PWM signal is constant in both types of PWM battery charge controllers. Moreover, the PWM waveform that is generated by the battery charge controllers may be considered constant over the recoding interval. The recording interval is defined as the time between records [42], and it is one minute. As shown later, the proposed technique must be used in PWM signals where at least one waveform parameter is known. In the case of series PWM battery charge controllers, the ‘low’ state of the current (i_A,H_) of the PV array is zero amps. Meanwhile, in the case of shunt PWM battery charge controllers, the ‘low’ state of the PV array voltage (v_A,L_) is zero volts.

### 3.1. Shunt Charge Controller—Estimated P_A,dc_ and E_A_

Different techniques to determine the duty factor and power were proposed by Williams et al. for shunt battery charge controllers [53]. It was observed that their techniques provided errors, which depended on the state of battery, irradiance, and loads. A high value of energy measurement error was observed when there was a dip in irradiance. Based on the techniques proposed by Williams et al., the sampling interval was selected according to the frequency signal and does not allow reconstruction of the PWM waveform. In this sense, in this paper, another approach was developed to estimate the array power and energy when the battery is in the PWM mode for shunt PWM battery charge controllers, in order to avoid dependency on irradiance, loads, and the SOC of the battery.

The ‘low’ state PV array voltage parameter is known in shunt BCC; this parameter corresponds to the short-circuit voltage of the PV array. Because of this, the first step in this case was to estimate the PV array voltage waveform’s parameters:

(3) $$v_{A,\mathrm{Average}}=\frac{1}{T}\times \int_{0}^{T}v\left(t\right)\mathrm{dt}\approx \frac{1}{T}\times \left(\int_{0}^{t_{\mathrm{ON}}}v_{A,H}\;\mathrm{dt}+\int_{t_{\mathrm{ON}}}^{t_{\mathrm{off}}}v_{A,L}\;\mathrm{dt}\right)=v_{A,H}\times d_{f,v}$$

(4) $$v_{A,\mathrm{rsm}}=\sqrt{\frac{1}{T}\times \int_{0}^{T}v{\left(t\right)}^{2}\mathrm{dt}}\approx \sqrt{\frac{1}{T}\times \left(\int_{0}^{t_{\mathrm{ON}}}v_{A,H}{}^{2}\mathrm{dt}+\int_{t_{\mathrm{ON}}}^{t_{\mathrm{off}}}v_{A,L}{}^{2}\mathrm{dt}\right)}=v_{A,H}\times \sqrt{d_{f,v}}$$

where v_A,Average_ and v_A,rsm_ are, respectively, the average voltage values of the PV array and the true root mean square voltage of the PV array. v_A,L_ represents the ‘low’ state of the voltage array waveform and v_A,H_ is the ‘high’ state of the voltage array waveform.

The duty factor can be obtained as:

(5) $$\{\begin{array}{c} v_{A,\mathrm{Average}}\approx v_{A,H}\times d_{f,v}\\ v_{A,\mathrm{RSM}}\approx v_{A,H}\times \sqrt{d_{f,v}\;} \end{array}\;d_{f,A,v}=\frac{v_{A,\mathrm{Average}}{}^{2}\left(A\right)}{v_{A,\mathrm{RSM}}{}^{2}\left(A\right)}$$

The ‘high’ state of the voltage can be estimated as:

(6) $$v_{A,H}=\frac{v_{A,\mathrm{Average}}\left(A\right)}{d_{f,v}}=\frac{v_{A,\mathrm{RSM}}{}^{2}\left(A\right)}{v_{A,\mathrm{Average}}\left(A\right)}$$

The PWM voltage waveform of a PV array can be defined as follows:

(7) $$v_{A}\left(t\right)=\{\begin{array}{cc} v_{A,H} & \mathrm{PWM}\;\mathrm{ON}\\ 0 & \mathrm{PWM}\;\mathrm{OFF} \end{array}$$

Likewise, the PWM array current waveform can be estimated:

(8) $$i_{A,\mathrm{Average}}=\frac{1}{T}\times \int_{0}^{T}i\left(t\right)\mathrm{dt}\approx \frac{1}{T}\times \left(\int_{0}^{t_{\mathrm{ON}}}i_{A,L}\;\mathrm{dt}+\int_{t_{\mathrm{ON}}}^{t_{\mathrm{off}}}i_{A,H\;}\mathrm{dt}\right)=i_{A,L}\times d_{f,v}+i_{A,H}\times \left(1-d_{f,v}\right)$$

(9) $$i_{A,\mathrm{RSM}}=\sqrt{\frac{1}{T}\times \int_{0}^{T}i{\left(t\right)}^{2}\mathrm{dt}}\approx \sqrt{\frac{1}{T}\times \left(\int_{0}^{t_{\mathrm{ON}}}i_{A,L}{}^{2}\mathrm{dt}+\int_{t_{\mathrm{ON}}}^{t_{\mathrm{off}}}i_{A,H\;}{}^{2}\mathrm{dt}\right)}=\;=\sqrt{\left(i_{A,L}{}^{2}\times d_{f,v}+i_{A,H\;}{}^{2}\times \left[\left(1-d_{f,v}\right)\right]\right)}$$

(10) $$i_{A,H\;}{}^{2}\times \left(1-d_{f,v}\right)-i_{A,H}\times 2\times i_{A,\mathrm{average}}\times \left(1-d_{f,v}\right)-i_{A,\mathrm{rsm}}{}^{2}\times \left(d_{f,v}\right)+i_{A,\mathrm{average}}{}^{2}$$

(11) $$i_{A,L}=\frac{i_{A,\mathrm{average}}-i_{A,H}\times \left(1-d_{f,v}\right)}{d_{f,v}}$$

where i_A,Average_ is the average current of the photovoltaic array, i_A,rsm_ represents the true root mean square current of the PV array, i_A,H_ and i_A,L_ are the ‘high’ state and ‘low’ state of the PV current respectively and d_f,i_ depicts the duty factor of PV array current.

In such a way, the PV array current can be defined as follows:

(12) $$i_{A}\left(t\right)=\{\begin{array}{cc} i_{A,L} & \mathrm{PWM}\;\mathrm{ON}\\ i_{A,H} & \mathrm{PWM}\;\mathrm{OFF} \end{array}$$

Once i_A,H_, v_A,H_, and d_f_ are obtained, PV array DC output power (P_A,dc_), when the BCC is in PWM mode, is given by:

(13) $$P_{A,\mathrm{dc}}=v_{A,H}\times i_{A,L}\times d_{f,v}$$

Moreover, the PV array DC output energy (E_A_) can be estimated as:

(14) $$E_{A}=\underset{k}{\sum }P_{A,\mathrm{dc}}\times \tau_{k}$$

where τ_k_ denotes the duration of the kth recording interval within a reporting period [42].

### 3.2. Series Charge Controllers—Estimated P_A,dc_ and E_A_

PV array DC output power and energy can be estimated if the four states of voltage and current waveforms (Table 1, series BCC), together with the d_f_, have been previously calculated. As indicated above, the ‘low’ state of current in a PV array is known in series PWM battery charge controllers; it has a value of zero. The other states of the waveform could be estimated following the technique developed in Reference [54] for series PWM battery charge controllers, where the first parameters to be estimated are the duty factor and the ‘high’ state current of a PV array. The duty factor parameter can be expressed as a function of the average and true rms array current:

(15) $$d_{f,i}=\frac{i_{A,\mathrm{Average}}{}^{2}\left(A\right)}{i_{A,\mathrm{RSM}}{}^{2}\left(A\right)}$$

The ‘high’ state of the current can then be estimated as:

(16) $$i_{A,H}=\frac{i_{A,\mathrm{Average}}\left(A\right)}{d_{f,i}}=\frac{i_{A,\mathrm{RSM}}{}^{2}\left(A\right)}{i_{A,\mathrm{Average}}\left(A\right)}$$

when the d_f,i_ and i_A,H_ parameters are obtained, the PWM current waveform of the PV array can be defined as follows:

(17) $$I_{A}\left(t\right)\approx \{\begin{array}{cc} i_{A,H} & \mathrm{PWM}\;\mathrm{ON}\\ 0 & \mathrm{PWM}\;\mathrm{OFF} \end{array}$$

Thereafter, v_A,H_ and v_A,L_ can be obtained:

(18) $$v_{A,H\;}{}^{2}\times \left(1-d_{f,i}\right)-v_{A,H}\times 2\times v_{A,\mathrm{average}}\times \left(1-d_{f,i}\right)-v_{A,\mathrm{rsm}}{}^{2}\times \left(d_{f,i}\right)+v_{A,\mathrm{average}}{}^{2}$$

(19) $$v_{A,L}=\frac{v_{A,\mathrm{average}}-v_{A,L}\times \left(1-d_{f,i}\right)}{d_{f,i}}$$

In this way, the PV array voltage can be defined as follows:

(20) $$V_{A}\left(t\right)\approx \{\begin{array}{cc} v_{A,L} & \mathrm{PWM}\;\mathrm{ON}\\ v_{A,H} & \mathrm{PWM}\;\mathrm{OFF} \end{array}$$

Similarly to shunt PWM battery charge controllers, PV array DC output power and energy in the regulation setpoint can be estimated as:

(21) $$P_{A,\mathrm{dc}}=v_{A,L}\times i_{A,H}\times d_{f,i}$$

(22) $$E_{A}=\underset{k}{\sum }P_{A,\mathrm{dc}}\times \tau_{k}$$

In short, electronic terms, such as the average and the root mean square voltage and current of a signal, are used in the proposed techniques for shunt and series PWM battery charge controllers. These can easily be obtained with inexpensive electronic circuits. Moreover, the former may be monitored using a sampling and recording interval of one minute. Using these values obtained previously, the first step to be accomplished is to determine the d_f_ of the PWM voltage (shunt) and the PWM array current (series) waveform. The second step is to estimate the ‘high’ state of PWM voltage (shunt) and current (series) waveform of the array and afterwards the ‘low’ and ‘high’ state of PWM current (shunt) or voltage (series) waveform of the array can be estimated. Finally, PV array DC output power and energy provided by the array in the PWM mode can be estimated. Different techniques to measure SAPV systems with a Pulse Width Modulation battery charge controllers are summarized in Table 2.

## 4. Experimental Set-Up

**Figure 4:**
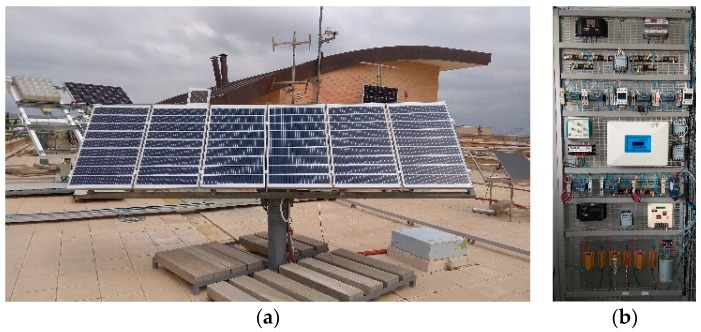
SAPV systems installed in the Higher Polytechnic School of Jaén. (**a**) PV generators and (**b**) BCCs, sensors, and traducers.

Two SAPV systems, their sensors, and a data logger system were installed in the Higher Polytechnic School of Jaén (latitude: 37 deg 46′00″ N and longitude 3 deg 47′0″ W), Figure 4. The monitoring system provided the data that were used to verify the monitoring techniques described above. In both systems, the modules are aligned to the south, and the tilt angle of the PV modules is 50° to the horizontal ground.

Each SAPV system has a PV array which is composed of two modules connected in parallel, a PWM battery charge controller, a 12 V 200 Ah lead–acid battery, and a load set with which different load profiles can be provided. In system #1, there is a series PWM battery charge controller, while system #2 uses a shunt PWM battery charge controller.

The PV array current (I_A_) was measured through calibrated shunt resistance (Table 3). On the other hand, the voltage parameter was measured directly by the DAS. These parameters were measured by high accuracy DAS, where the sampling interval was set at 200 times the maximum frequency of the signal to be measured, which follows the recommendation of Annex A of the IEC 61724-1 standard. The device used to measure the parameters was a cDAQ-9172 chassis with two modules to collect the parameters: 2×NI 9229. It needs to be highlighted that this hardware was used to verify the proposed monitoring technique. Table 3 also shows the measured and recorded parameters, as well as those of the National Instruments (NI) modules used in the DAS.

**Figure 5:**
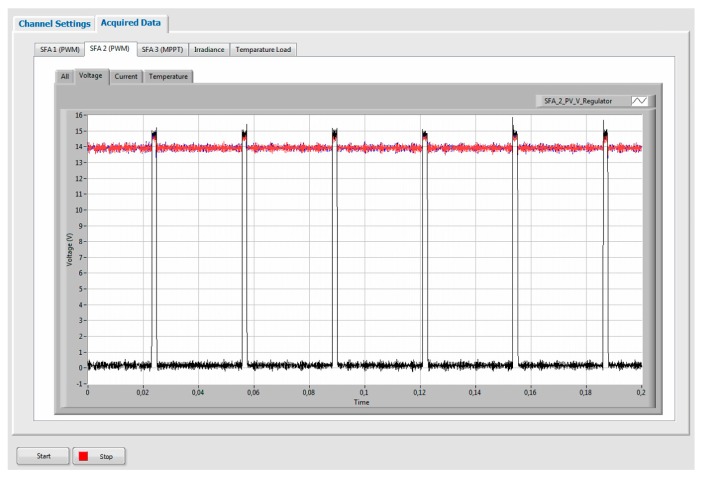
The graphical user interface for SAPV systems monitoring used in the experiment.

**Figure 6:**
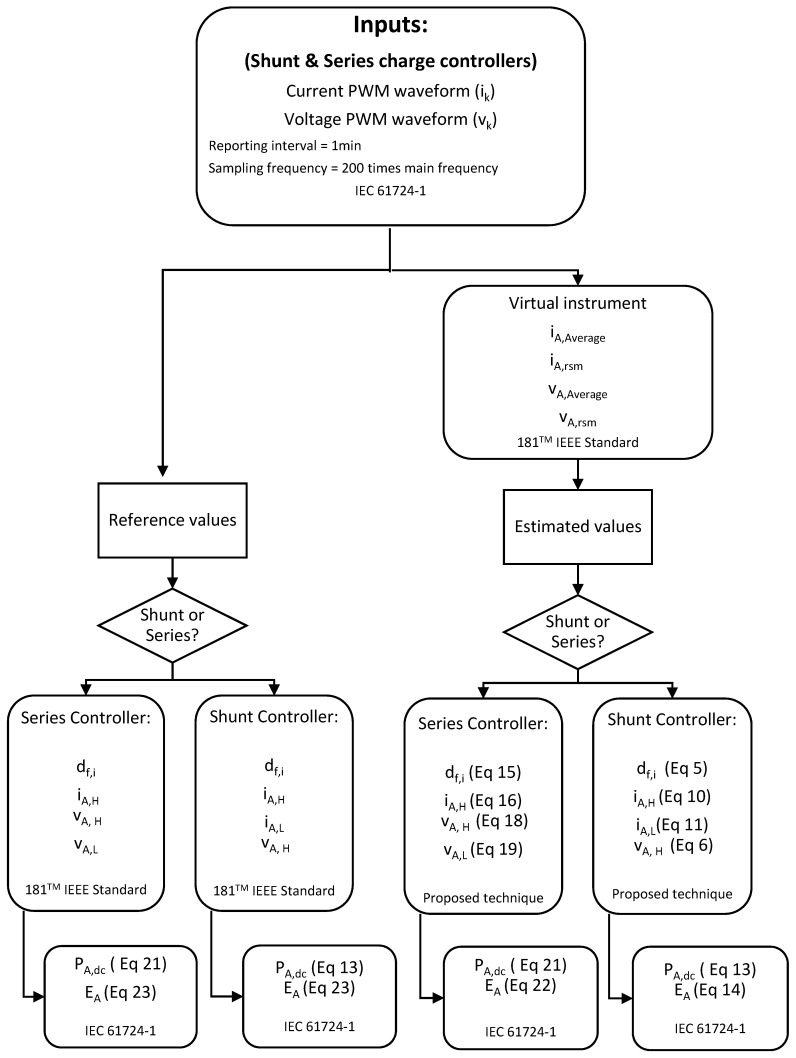
A flowchart of the experimental setup to validate the proposed monitoring techniques using real PWM signals from two SAPV systems (series and shunt PWM charge controllers).

A graphical user interface (GUI) (Figure 5) developed in LabVIEW@ was used to measure array currents and voltage, following the recommendations of Annex A of the IEC 61724-1 standard. The application allowed communication with the DAS and recorded the monitored data. In this sense, the real PWM waveforms from two types of charge controllers were obtained. The high state, the low state, and the duty factor of current and voltage waveforms were obtained and constituted the reference values. Mean current, mean voltage, true root mean square current, and voltage of the real PWM waveforms ertr calculated with a virtual tool developed by Matlab^®^, and they were be used as inputs for the proposed techniques. All these parameters were estimated according to the 181^TM^ IEEE standard on transitions, pulses, and related waveforms [43]. Later, mean current, mean voltage, true root mean square current, and voltage were processed according to the proposed techniques to estimate the high state, the low state, and the duty factor of current and voltage waveforms. These values constituted the estimated values. Furthermore, array DC power and energy were calculated by using reference and estimated values (Figure 6). Finally, the collected data were used to calculate the error between the different parameters, reference and estimated, in order to analyze and validate the aforementioned techniques.

(23) $$E_{A}=\underset{k}{\sum }i_{k}\times v_{k}$$

where k denotes the number of samples in a reporting period, and i_k_ and v_k_ are the current and voltage values in the kth sample.

## 5. Results and Discussion

The measurement campaign was carried out from October to November 2017. Although the recorded data correspond to complete days, the array data collected when the battery charge controller operated at PWM mode have only been considered in order to restrict the analysis to PWM signals.

The aforementioned analysis was focused on the estimation of the duty factor, low and high states of the current, low and high states of voltage, array power dc, and daily array energy dc in both types of battery charge controller: shunt and series. There was no set of validation techniques which could be used for all modeling; in fact, plots can be a good diagnostic alternative where there are measured and estimated parameters [55]. Therefore, three different statistical metric indicators were used in order to provide robust validation. These parameters have been used in other similar studies [11,56,57]. In order to validate the proposed technique, statistical metric indicators were used: normalized root mean square error (NRMSE), Equation (24), normalized mean bias error (NMBE) Equation (25), mean absolute percentage error (MAPE), Equation (26), and percentage error (PE) Equation (27).

(24) $$\mathrm{NRMSE}\left(\%\right)=100\times \frac{\sqrt{\frac{1}{N}\times \sum_{i=1}^{N}{\left(X_{\mathrm{estimated}}-X_{\mathrm{ref}}\right)}^{2}}}{{\bar{X}}_{\mathrm{ref}}}$$

(25) $$\mathrm{NMBE}\left(\%\right)=100\times \frac{\frac{1}{N}\times \sum_{i=1}^{N}\left(X_{\mathrm{estimated}}-X_{\mathrm{ref}}\right)}{{\bar{X}}_{\mathrm{ref}}}$$

(26) $$\mathrm{MAPE}\left(\%\right)=\frac{100}{N}\times \overset{N}{\underset{i=1}{\sum }}\left|\frac{\left(X_{\mathrm{estimated}}-X_{\mathrm{ref}}\right)}{X_{\mathrm{ref}}}\right|$$

(27) $$\mathrm{PE}\left(\%\right)=100\times \frac{\left(X_{\mathrm{estimated}}-X_{\mathrm{ref}}\right)}{X_{\mathrm{ref}}}$$

where X represents the parameter considered and N is the number of samples.

The sampling frequency was selected according to the 61724-1 standard [42]. In this sense, reference values are the collected samples at this frequency, while estimated values were obtained through the proposed technique. From PWM waveforms, the corresponding average and true RMS values were obtained considering a sampling and recording interval of one minute. The collected data were used to estimate the duty factor and the different state levels, through the techniques described in Section 3. Afterwards, reference and estimated values were compared. Current and voltage parameters were measured with shunt resistors and directly by DAS, respectively.

The results obtained for all samples after applying Equations (24)–(26) are presented in Table 4 and Table 5.

### 5.1. Shunt Charge Controller

**Figure 7:**
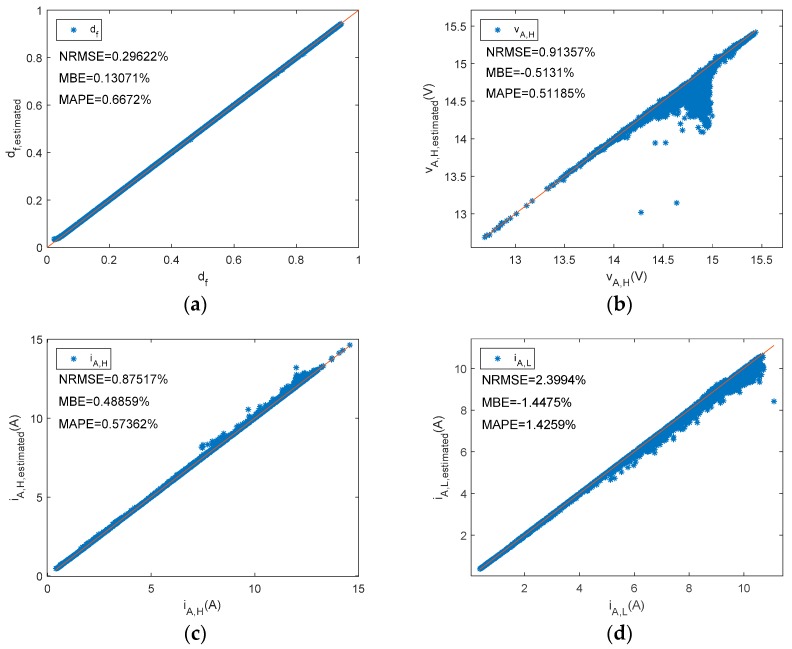
A shunt PWM battery charge controller reference versus estimated parameters. (**a**) d_f_, (**b**) v_A,H_, (**c**) i_A,H_, (**d**) i_A,L_.

**Figure 8:**
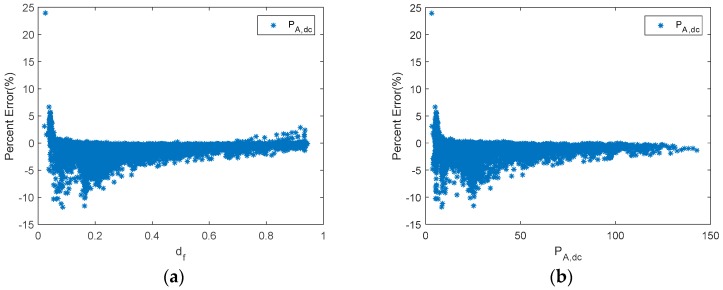
A shunt PWM battery charge controller. (**a**) Percent error of P_A__,dc_ versus d_f_. (**b**) Percent error of P_A__,dc_ versus P_A__,dc_.

**Figure 9:**
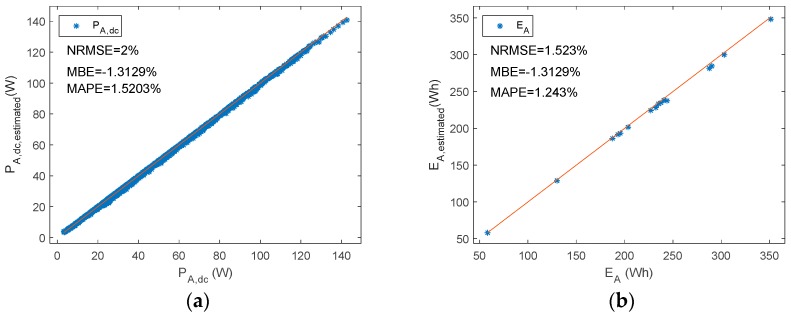
A shunt PWM battery charge controller reference versus estimated parameters (**a**) P_A__,dc_ (**b**) E_A._

Figure 7, Figure 8 and Figure 9 plot estimated state levels versus reference levels. d_f_, v_A,H_, i_A,H_, and i_A,L_ were estimated based on Equations (5), (6), (10), and (11), respectively. Equations (13) and (14) were used to estimate power PV array DC output power and energy, respectively. In this type of BCC, the low state of the array voltage should be zero volts. There is also an appropriate range in Figure 7b, to enhance visual perception.

**Figure 10:**
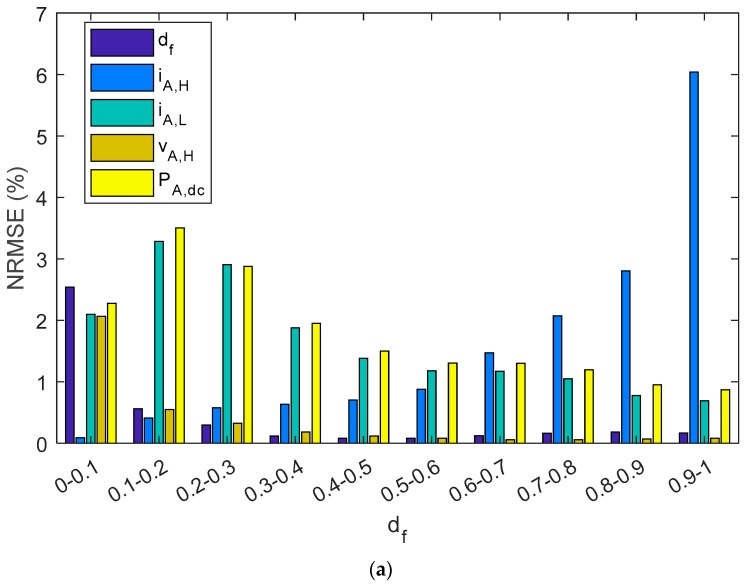

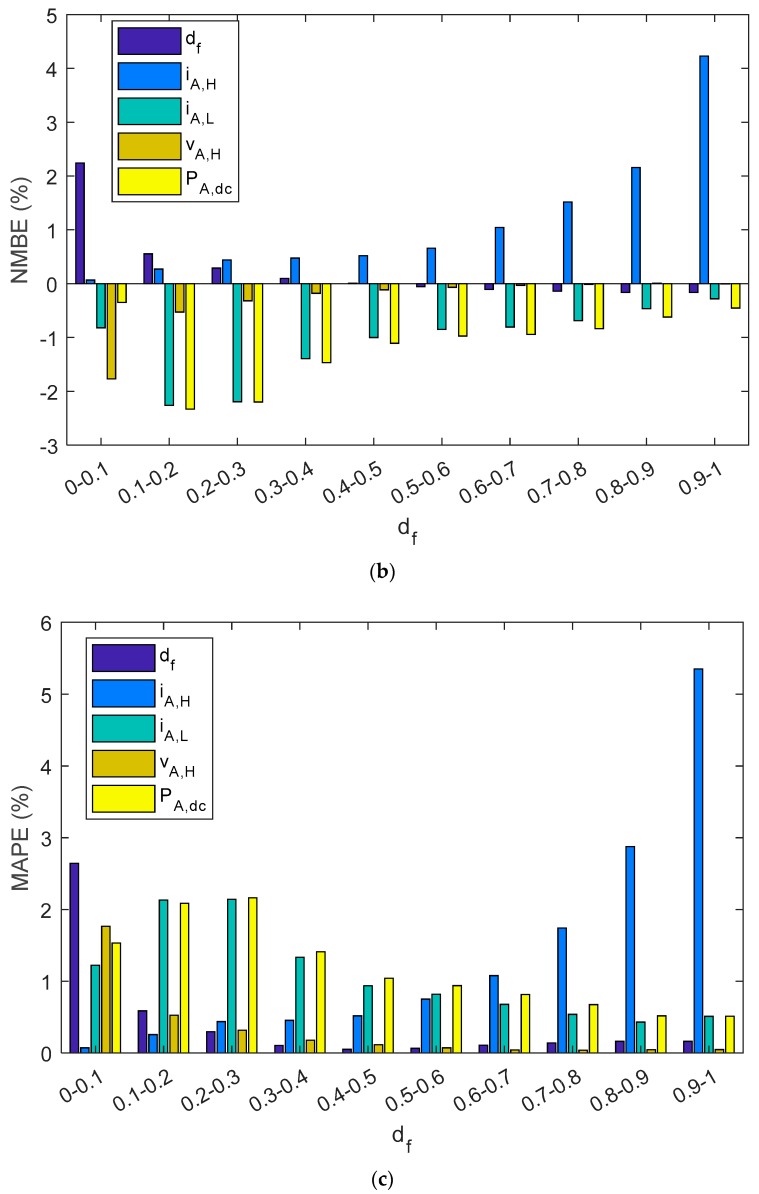
A shunt PWM battery charge controller bar diagram shows: (**a**) NRMSE, (**b**) NMBE, (**c**) MAPE. The results have been grouped in ten intervals of the duty factor.

Figure 10 summarizes the NMRSE, NMBE, and MAPE of shunt BCC, where the results have been divided into ten intervals of the duty factor. In the study of shunt charge controllers, and for all the parameters considered, NRMEs were below 2.4%. Likewise, NMBEs ranged between −1.5% and 0.5%, and the MAPE was lower than 1.6%. On the other hand, as is shown in Figure 10a, the NRME had the highest value for i_A,H_ when the d_f_ is was the range of 0.9–1.0, and it was 6%. Nevertheless, the NRME for i_A,H_ decreased when the d_f_ decreased to negligible values (<1% when d_f_ < 0.6). On the other hand, the other parameters, d_f_, v_A,H_, i_A,L_, and P_A,dc_ decreased when the d_f_ increased. The MBEs and MAPEs had the same tendency as NRMEs, but the NMBE was lower than 4.2% and the MAPE was below 5.4%. Therefore, P_A,dc_ had the biggest NRME, NMBE, and the MAPE when the d_f_ approached zero. Nevertheless, very low duty factors were reached when there was a high battery SOC; therefore, the estimated energy from the PV array that goes to the battery may be negligible.

As has been shown, the error in the estimation of the different parameters depends on the duty factor. Williams et al. proposed different techniques in order to estimate P_A,dc_ in shunt BCC [53]. The results of their proposals also showed the PE dependency on the duty factor. In this sense, when the duty factor was lower than 0.6, the PE was higher than 5%. Moreover, when the duty factor was lower than 0.3 and higher than 0.2, the percent error rate was between 7.5% and 17.5%. However, there is no information about PE when duty factor is lower than 0.2.

On the other hand, the PE obtained using the proposed monitoring techniques is shown in Figure 8, which, throughout most of the measurement range, provided slight differences between estimated and reference values. Moreover, results are shown considering duty factors higher than 0.02. As is shown in Figure 8, when the duty factor was higher than 0.35, the PE was lower than 5%. On the other hand, when the duty factor was higher than 0.2 and lower than 0.35, the PE had a value lower than 8%. Meanwhile, the PE with a value of duty factor lower than 0.2 had the higher value, and the values were lower than 12%. In addition, they were generally lower than 10% when the duty factor was lower than 0.2. As can be seen, the results are considerably better than those obtained by Williams et al. Moreover, it should be taken into account that the mean absolute percentage error (MAPE) was 3.6% in the duty factor range 0–0.1, and 2.1% in the duty factor range 0.1–0.2. It must be outlined that very low duty factors were reached when there was a high battery SOC. Therefore, the estimated array power to battery in this case, may be negligible. Thus, the estimated energy from the PV array will be almost unaffected.

### 5.2. Series Charge Controller

**Figure 11:**
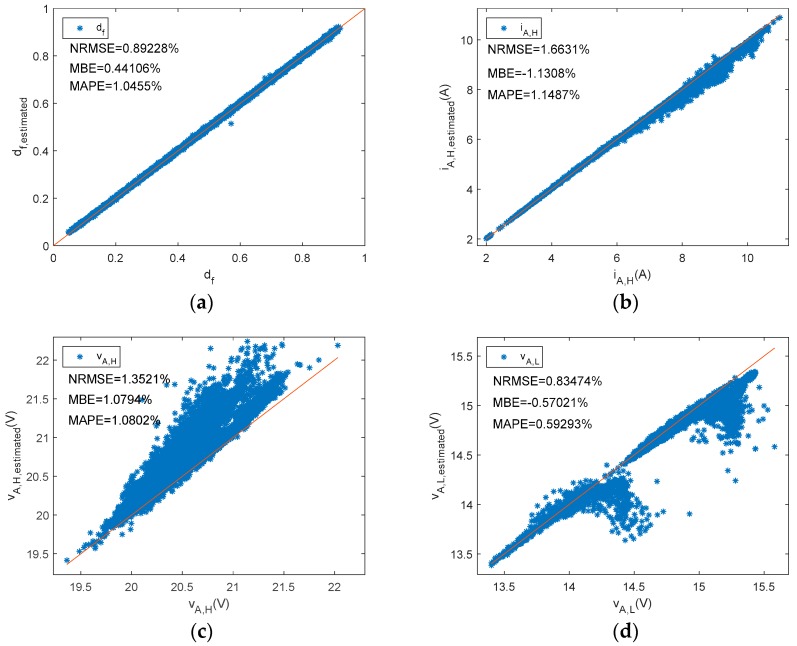
A series PWM battery charge controller. Reference versus estimated parameters. (**a**) d_f_, (**b**)i_A,H_, (**c**) v_A,H_, (**d**) v_A,L_.

Similarly, Figure 11 plots estimated state levels versus reference ones. Meanwhile, for the series BCC d_f_, i_A,H_, v_A,H_, and v_A,L_ were estimated using Equations (15), (16), (19), and (20), respectively. The low state of the array current should be zero amps for this series BCC. In order to enhance visual perception, an appropriate range has been selected in Figure 11c,d.

**Figure 12:**
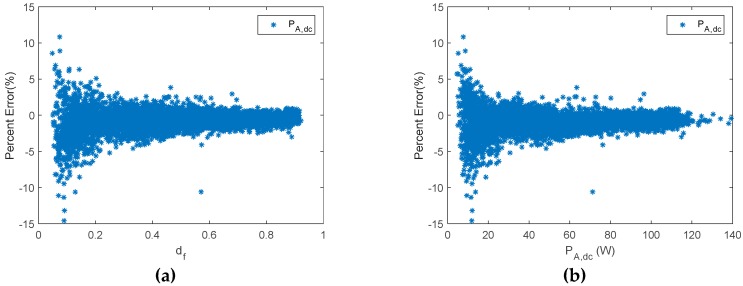
A series PWM battery charge controller. (**a**) Percent error of P_A__,dc_ versus d_f_. (**b**) Percent error of P_A__,dc_ versus P_A__,dc_.

**Figure 13:**
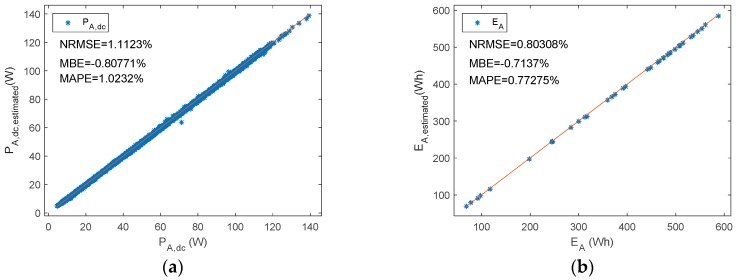
A series PWM battery charge controller. Reference versus estimated parameters. (**a**) P_A,dc_ (**b**) E_A_.

Figure 12 shows the PE of P_A,dc_ versus d_f_ and P_A,dc_, while Figure 13 plots estimated P_A,dc_ and E_A_ versus reference ones.

**Figure 14:**
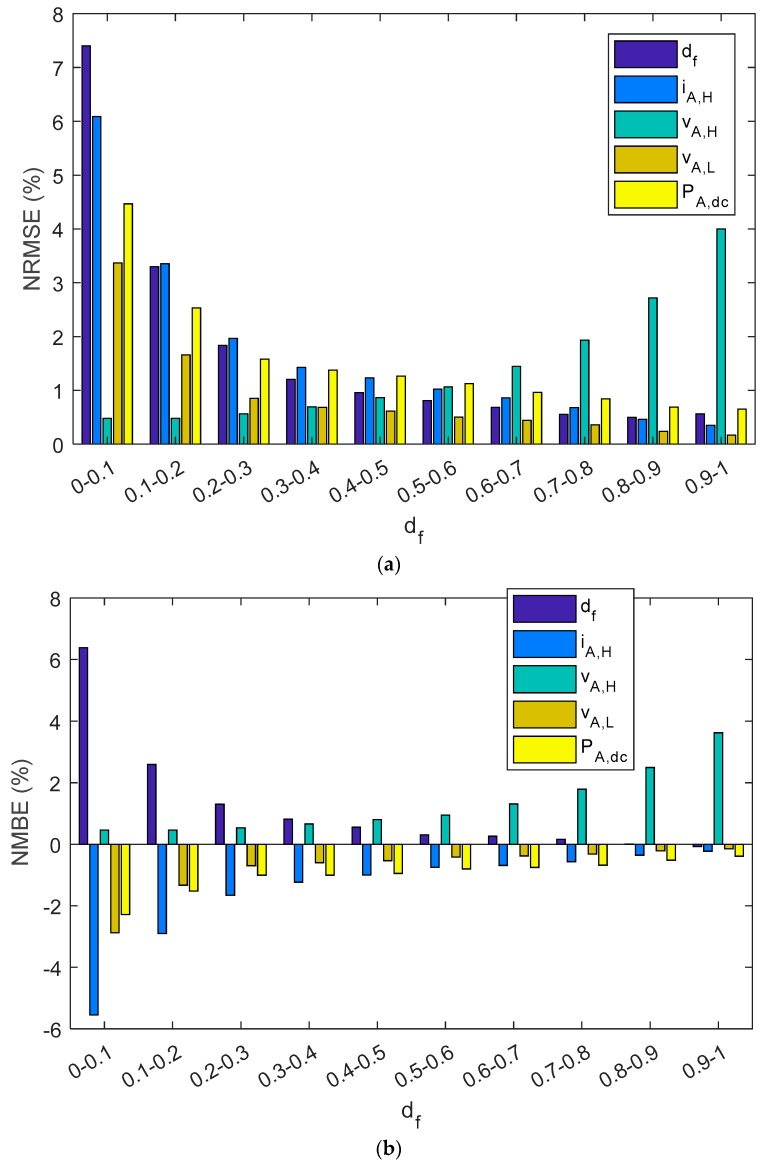

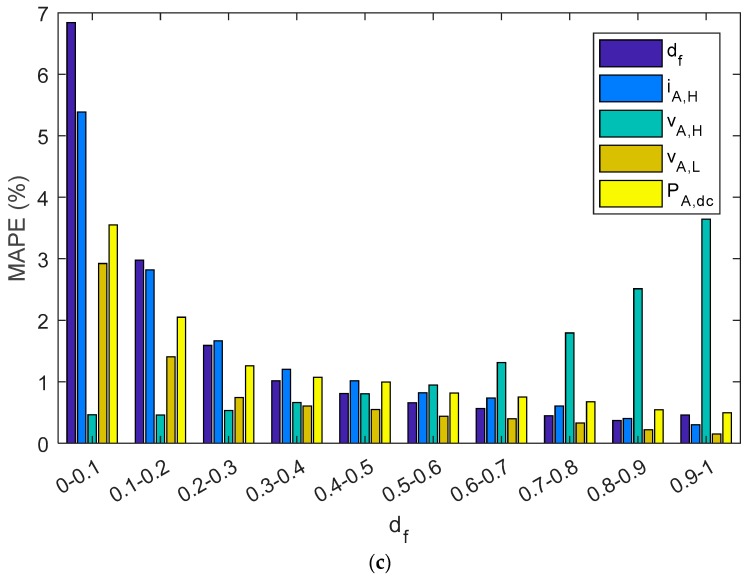
A series PWM battery charge controller bar diagram shows: (**a**) NRMSE, (**b**) NMBE, (**c**) MAPE. The results have been grouped in ten intervals of the duty factor.

As can be observed in Table 5 and Figure 14, estimated parameters in the series charge controllers slightly differed from reference values as NRMEs was lower than 1.7%, NMBE ranged between −1.15% and 1.1%, and MAPE was below 1.15%. In addition, when parameters were grouped into different intervals as a function of the duty factor, the NRME and MAPE became a little bit higher as the d_f_ diminishes for d_f_, i_A,H_, v_A,L_, and P_A,dc_, but always remained below 7.5%. As with shunt BCCs, when there is a low d_f_ (i.e., battery has a high SOC), the estimated array power and energy may be negligible. NMBEs had the same tendency for these parameters, but had a positive value for the d_f_ and v_A,H_, and negative values for i_A,H_, v_A,L_ and P_A,dc_. This implies an overestimation for the d_f_ and v_A,H_ and a sub-estimation of the i_A,H_, v_A,L_, and P_A,dc_. On the other hand, when v_AH_ had a higher duty factor value, the NMBE, NRME, and MAPE were higher. As can be observed in Equation (1), the duty factor corresponding to the voltage waveform was proportional to the subtraction of one minus the duty factor of the current waveform. Thus, v_A,H_ is the voltage when the battery and the PV array are not connected (State level #1).

## 6. Conclusions

Various issues should be considered when monitoring array current and voltage signals of SAPV systems with PWM battery charge controllers in order to estimate P_A,dc_ and E_A_. The sampling frequency, the use of simultaneous channels, and the large amount of data collected, which have all been highlighted throughout this paper, can be obtained with advanced devices, although this choice could be inappropriate due to the relatively low cost of most SAPV systems.

In this paper, new and simple monitoring techniques for SAPV with shunt and series PWM battery charge controllers have been developed. These techniques can estimate P_A,dc_ and E_A_ without using complex and sophisticated DASs. For this purpose, it is necessary to estimate the d_f,i_ of the PWM array current waveform for series PWM battery charge controllers, or the d_f,v_ of the PWM array voltage waveform for shunt PWM battery charge controllers. Moreover, i_A,H_, i_A,L_, v_A,H_, and v_A,L_ parameters can be estimated by measuring the average and the true mean square values of the signals. From the true rsm and average values, the techniques shown here allow estimation of P_A,dc_ and E_A_, considering d_f_ and one of the previous values (average or true mean square). Only unsophisticated DAQ devices, together with transducers, sensors, or electronic circuits designed ad hoc, are needed. Moreover, the different estimated parameters will permit the reconstruction of the PWM signals.

The equations developed were obtained considering “clean” PWM waveforms. However, the “real” waveforms provided by charge controllers may have overshoot, undershoot, ringing, or positive and negative transition durations, so the validity of the proposed techniques was checked in real operation with different values of the duty factor. In order to validate the proposed techniques, the array PWM signals corresponding to two SAPV systems with a series and a shunt PWM battery charge controllers were monitored, respectively, through a sophisticated DAS with a sampling and recording interval in compliance with the Annex A of the IEC 61724, and through their average and true RMS values, considering only a sampling and recording interval of one minute.

Estimated parameters in series charge controllers slightly differed from reference values, as NRMEs were lower than 1.7%, the NMBE ranged between −1.15% and 1.1%, and the MAPE was below 1.15%. Similarly, in shunt battery charge controllers NRMEs were below 2.4%. Likewise, NMBEs ranged between −1.5% and 0.5% and the MAPE was lower than 1.6%. In accordance with the results obtained, the proposed techniques allow calculation of PV array DC output power (P_A,dc_) and PV array DC output energy (E_A_), avoiding the use of complex and expensive DASs.

Once the monitoring techniques have been validated, the method shown here can be implemented in different ways using basic and simple DAQ devices, together with average and true rms transducers. Moreover, the DAQ device can even be substituted by open hardware–software platforms, and the transducers can be substituted by electronic circuits designed ad hoc to obtain average and true rms values. Any of these different choices can help to provide P_A,dc_ and E_A_ in a simple way. Further investigation should be addressed in order to design prototypes with open source hardware/software which allow application of the techniques shown here.

Furthermore, the techniques here shown to monitor the array power in SAPV systems with PWM charge regulators may be also used to monitor PWM signals in other applications, such as the speed control of DC motors and the brightness adjustment of light emitting diodes (LED), which may be controlled by PWM dimming as it allows adjustment of the average LED current [58].

## Figures and Tables

**Table 1:** State levels of pulse width modification (PWM) battery charge controllers.

BCC	State Level	PV Array Current	PV Array Voltage
Series	#1	High state (i_A,H_)	Low state (v_A,L_) or open circuit voltage (V_OC_)
	#2	Low state (i_A,L_)	High state (v_A,H_) or battery voltage (V_S_)
Shunt	#1	Low state (i_A,L_)	High state (v_A,H_) or battery voltage (V_S_)
	#2	High state (i_A,H_) or short circuit current (I_SC_)	Low state (v_A,L_)

**Table 2:** Monitoring SAPV systems with pulse width modulation battery charge control.

Ref:	Technique:	Summary
[53]	Power monitoring (voltage and current)	The technique only applies to shunt PWM BCCs. High errors are reported, which depend on the battery SOC.
[10,11,12]	Current monitoring	The techniques apply to series and shunt PWM charge controllers. It provides a proper performance analysis based on charge parameters. However, it does not permit performance comparisons with SAPV systems with MPPT charge controllers, as the latter are based on energy parameters. Array current and voltage PWM signals cannot be reconstructed.
[54]	Current and voltage waveforms	The technique only applies to series PWM BCCs. It provides only the four state parameters of the current and voltage waveforms. It also provides the PWM duty factor.
Proposed monitoring techniques	Power monitoring (voltage and current)	The technique applies to either series or shunt PWM BCC. Array current and voltage PWM signals can be reconstructed, avoiding complex and sophisticated DAQ devices. It provides the PWM duty factor. It monitors array power array (P_A,dc_) with a negligible error. It provides a proper analysis based on energy parameters (E_A_).

**Table 3:** Measured and recorded parameters, transducers, and DAS NI modules.

Measured and Recorded Parameters	Symbol (Unit)	Transducers and Sensor	DAS NI Module
Array current	I_A_ (A)	Shunt (25 A, 150 mV)	NI 9229 Calibrated
Array output voltage	V_A_ (V)	-	

**Table 4:** A shunt PWM battery charge controller. Normalized root square mean error (NRMSE), normalized mean bias error (NMBE), and mean absolute percentage error (MAPE) between reference versus estimated parameters (Equations (24)–(26)).

Parameter	NRMSE (%)	NMBE (%)	MAPE (%)
d_f_	0.2962	0.1307	0.6672
v_A,H_	0.9136	−0.5131	0.5119
i_A,H_	0.8752	0.4886	0.5736
i_A,L_	2.3994	−1.4475	1.4259
P_A,dc_	2.0000	−1.3129	1.5203
E_A_	1.5230	−1.3129	1.2430

**Table 5:** A series PWM battery charge controller. Normalized root mean square error (NRMSE), normalized mean bias error (NMBED), and mean absolute percentage error (MAPE) between reference versus estimated parameters (Equations (24)–(26)).

Parameter	NRMSE (%)	NMBE (%)	MAPE (%)
d_f_	0.8923	0.4411	1.0455
i_A,H_	1.6631	−1.1308	1.1487
v_A,H_	1.3521	1.0794	1.0802
v_A,L_	0.8347	−0.5702	0.5929
P_A,dc_	1.1123	−0.8077	1.0232
E_A_	0.8031	−0.7137	0.7728

## Abbreviations

BCC	Battery charge controller
BOS	Balance of systems
DAS	Data acquisition systems: sensors, transducers, electronic circuit, data acquisition devices or data acquisition boards, computer, etc.
DAQ	Data acquisition device
DC	Direct current
d_f_	Duty factor
d_f,i_	Duty factor of PV array current waveform
d_f,v_	Duty factor of PV array voltage waveform
E_A_	PV array DC output energy
I_A_ (A)	Array current
i_A,average_	Average current of the PV array
i_A,H_	‘High’ state of current of PV array
i_A,L_	‘Low’ state of current of PV array
i_A,rsm_	True root mean square current of the PV array
I_L_ (A)	Load current
I_S_ (A)	Battery current
I_SC_ (A)	Short circuit current
IEC	International Electrotechnical Commission
MAPE	Mean absolute percentage error
MPPT	Maximum power point tracker
NMBE	Normalized mean bias error
NRMSE	Normalized root mean square error
PV	Photovoltaic
P_A,dc_	PV array DC output power
PWM	Pulse width modulation
SAPV	Stand-alone photovoltaic
SHSs	Solar home systems
SOC	State-of-charge
V_A_ (V)	Array output voltage
v_A,average_	Average voltage of the PV array
v_A,H_	‘High’ state of voltage of PV array
v_A,L_	‘Low’ state of voltage of PV array
v_A,rsm_	True root mean square voltage of the PV array
V_L_ (V)	Load voltage
V_OC_	Open circuit voltage
V_S_ (V)	Battery voltage
